# Toxic effect of *Tropaeolum majus* L. leaves on spermatogenesis in mice

**DOI:** 10.5935/1518-0557.20180035

**Published:** 2018

**Authors:** Layasadat Khorsandi, Ali Akbar Oroojan

**Affiliations:** 1 Department of Anatomical Sciences, Faculty of Medicine, Cellular and Molecular Research Center, Ahvaz Jundishapur University of Medical Sciences, Ahvaz, Iran; 2 Department of Physiology, Faculty of Medicine, Student Research Committee, Ahvaz Jundishapur University of Medical Sciences, Ahvaz, Iran

**Keywords:** Spermatogenesis, *Tropaeolum majus* L., toxicity, testis, mouse

## Abstract

**Objective:**

To evaluate the hydroethanolic extract toxicity, obtained from *Tropaeolum majus* L. (TM) on mouse testicular tissue.

**Method:**

In this experimental study, we used 32 male NMRI mice. The experimental groups received 75, 375 and 750 mg/kg of TM extract, respectively. Twenty-four hours after the last experimental day, serum samples were collected for hormonal measurement. Then, the cauda of epididymis and testis were removed for sperm count and histopathological assessments.

**Results:**

Testosterone serum and testicular levels decreased in 750 mg/kg in the treated group when compared to the control animals (1.65±0.25; *p*=0.041 and 98.83±8.67; *p*=0.034 respectively). Histopathological criteria such as epithelial vacuolization (9.3±1.1; *p*=0.034), sloughing (4.3±0.4; *p*=0.027) and detachment (12.2±0.9; *p*=0.031) of germ cells were significantly increased in 750 mg/kg in the treated mice. In addition, there were no significant changes in histopathological criteria; sperm head numbers, Johnsen's scoring, and morphometry assessments were carried out in the 75 and 375 mg/kg treated mice. At the dose of 750 mg/kg, the seminiferous tubule diameter (193.2±4.6; *p*=0.019), seminiferous epithelium height (139.2±5.1; *p*=0.023), and maturation arrest were significantly decreased in this group.

**Conclusion:**

In conclusion, TM extract has toxic effects on the mouse testicular tissue in high doses. Hence, we recommend caution concerning its consumption by patients with reproductive problems.

## INTRODUCTION

Spermatogenesis is dependent on hormonal stimulation and on dynamic interactions between the Sertoli cells and the germ cells of the seminiferous epithelium. This complex process produces spermatozoa in the testis (de França *et al.,* 1993; Boekelheide *et al.,* 2000). Sertoli cells generate a specialized microenvironment for the development and viability of resident germ cells by secreting hormonal and nutrition factors into the adluminal compartment. Moreover, these cells provide an efficient paracrine signaling mechanism and physical support for them (Cheng & Mruk, 2002). Some toxic agents disrupt spermatogenesis through an intricate regulation and cellular interactions that induce testicular tissue damage (Boekelheide *et al.,* 2000).

The use of medicinal plants for the treatment of diseases usually comes from the belief that they present low toxicity, because they are "natural herbs". However, treatments with medicinal plants, as well as conventional medication, may cause adverse effects and drug interactions (Seef, 2007; Jordan *et al.,* 2010). Herbal toxicity, as an important issue, induces a serious threat to human health (Chen *et al.,* 2011).

*Tropaeolum majus* L. (TM) is a medicinal plant used in folk medicine, to treat several diseases. Its leaves are used to treat several diseases, including cardiovascular disorders, urinary tract infections, asthma and constipation (Gomes *et al.,* 2012). Even though several studies proved its therapeutic effects (Gasparotto *et al.,* 2011; Koriem *et al.,* 2010; Butnariu & Bostan, 2011; Garzón & Wrolstad, 2009), only a few toxicological studies with TM can be found in literature reviews (Gomes *et al.*, 2012; Gasparotto *et al.*, 2009). Therefore, it is necessary to evaluate its safety. Hence, in this study we assessed the toxic effect of TM extract on mice spermatogenesis.

## MATERIALS AND METHODS

### Animals

In this experimental study, thirty-two healthy and adult male NMRI (Naval Medical Research Institute) mice (6-8 weeks old, 25-30 g) were used. The animals were obtained from the Ahvaz Jundishapur University of Medical Sciences, Experimental Research Center, and this study was approved by the ethics committee of the Jundishapur University with ethics' committee grantee No. (IR.AJUMS.REC.1395.417). After one week of acclimatization, the animals were kept in polycarbonate cages with corncob bedding in 20±4°C temperature with a 12h light/12h dark cycle and 50±5°C humidity. Those conditions were maintained until the end of the experiment. Tap water and commercial chow (pellet) were given ad libitum.

### Experimental design

The mice were randomly divided into 4 groups (n=8 animals in each group). TM extract was dissolved in normal saline and gavaged (0.5mL) at the doses of 75, 375, and 750 mg/kg (Gomes *et al.,* 2012) for 35 consecutive days. The control group received only normal saline by the gavage method for 35 consecutive days. One day after the final administration, the mice were slaughtered and their testicles were dissected and weighed. The right testis from each animal was fixed in 10% formalin. The samples were embedded in paraffin, sectioned (5µm) and stained with hematoxylin and eosin (H&E) for histopathology, Johnsen's scoring, and morphometric studies. The left testis was homogenized to count testicular sperm heads.

### Extract preparation

Fresh TM leaves were obtained from the Ahvaz city, Iran, in 2016 and scientifically approved by the Faculty of Pharmacy of the Ahvaz Jundishapur University of Medical Sciences. The leaves were air-dried in an oven at 40°C for 4 days and the resulting dry plant was cut and pulverized. This plant material was macerated for 7 days using 90% ethanol as solvent. A rotary vacuum evaporator under reduced pressure (Lourenço *et al*., 2012) then eliminated the solvent. Finally, the yield ratio of the extract was 9.8 g/100mL.

### Histopathology

Six slides of histopathological alterations for each animal revealed signs of germ cell degeneration such as detachment (cohorts of spermatocytes breaking off from the seminiferous epithelium); sloughing (release of clusters of germ cells into the lumen of the seminiferous tubule) and vacuolization (empty spaces in the seminiferous tubules). The average percentage of normal and regressed tubules was determined for each treatment (Johnson, 2014).

### Morphometry

We use a graticule of a calibrated linear scale in the 10× eyepiece of the Leitz microscope with an objective lens 40× to assess the diameters of the seminiferous tubules and the seminiferous epithelium height. Only circular and near circular tubules were measured (Ma et al., 2000; Neves et al., 2002) . We assessed 150 tubules for each mouse. The primary volume (V primary) was measured by using the immersion method as previously described (Habert & Picon, 1982).

### Assessment of spermatogenesis

We used the Johnsen scoring method, a simple method for spermatogenesis assessment, to grade germinal epithelium maturity (Johnsen, 1970). One hundred tubules were assessed and each tubule was given a score ranging from 1 to 10. The complete inactive tubules were scored as 1 and those with maximum activity (at least five or more spermatozoa in the lumen) were scored as 10.

### Testicular sperm count

We assessed testicular sperm head numbers to evaluate the numbers of mature elongate spermatids in the testis. Briefly, mouse testes were homogenized in an 8 ml solution solution of 0.9% NaCl and 0.05% Triton X-100, and sperm heads were counted using a hemocytometer (Blazak *et al.,* 1993). Each sample was counted four times and averaged. To minimize error, the count was repeated at least five times for each mouse, by 2 or 3 examiners.

### Testosterone measurement

Twenty-four hours after the last extract administration, the mice were anesthetized and blood samples were collected in a heparinized centrifuge. All samples were centrifuged at 3500 rpm for 20 minutes to obtain serum. The serum samples were kept at −80°C until hormonal measurements were performed. Serum testosterone concentration was measured by the enzyme-linked immunosorbent assay (ELISA) method with commercial assay kits (DRG Instruments GmbH, Germany), and the hormone detection sensitivity per assay tube kit was 0.287 nmol/L (Muselin *et al.,* 2016). Testosterone was also extracted from testes as previously described (Ling *et al.,* 2008). Briefly, the testes were homogenized by sonication and centrifuged at 5900 × *g* for 5 min. The supernatant was combined with an equal volume of ethyl acetate, and the organic phase was dried under a stream of N2 gas at room temperature and reconstituted in 1× PBS. The ELISA test estimated the concentration of testosterone by this procedure.

### Statistical analysis

The data were statistically analyzed using the SPSS software (version 15; SPSS Inc., Chicago, Ill) with one-way ANOVA followed by Post hoc LSD test. Values were presented as the mean ± standard deviation (SD). *p*<0.05 was considered statistically significant.

## RESULTS

### Histological changes

**Figure 1:**
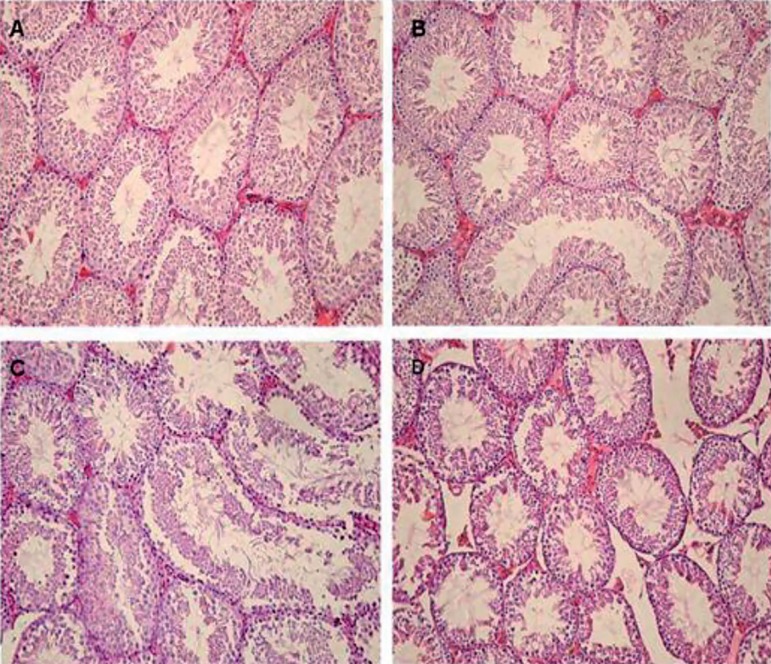
Light microscopy of cross sections of H & E stained testis from Control and Experimental groups. (A) Control testis: normal architecture of the seminiferous tubules is found. (B) 75mg/kg group, the tubules show normal spermatogenesis. (C) 375mg/kg group, there are histopathological changes including detachment, sloughing, and vacuolization. (D) Tubules show varying degrees of histopathological changes, and an increase in interstitial spaces between seminiferous tubules due to reduction in tubule diameter. (Magnifications: 40×).

Testicular sections from control animals showed a low incidence of detached or vacuolized seminiferous tubules (Figure 1A). The 75 mg/kg group demonstrated a normal architecture of the seminiferous tubules and intact germinal epithelium (Figure 1B). There was no significant difference in the histopathology criteria between the 75mg/kg and the Control groups. The 375mg/kg group had low degrees of germ cell degenerative changes, ranging from loss of elongated spermatids, disorganization of germ cell layers, detachment and sloughing to vacuolization of the seminiferous tubules (Figure 1C). In the 750mg/kg group (Figure 1D) all histopathology criteria such as detachment (12.22±0.91; *p*=0.031), sloughing (4.29±0.41; *p*=0.027) and vacuolization (9.35±1.12; *p*=0.034) were significantly decreased, when compared to the Control group (1.01±0.22; 0.63±0.04; 1.10±0.4; respectively). Percentages of normal tubules were significantly decreased in the 750 mg/kg treated mice(74.21±0.45; *p*=0.001) compared to control animals (97.32±1.41). Mainly, the Sertoli cells showed large basal vacuoles in their cytoplasm in the 750mg/kg group. The TM did not affect the Leydig cells. Table 1 shows the results obtained from the histopathological evaluations following TM treatment.

**Table 1:** Testis histopathology assessments for the Control and Experimental groups.

Groups	Percentage of tubules			
	Normal	Detached	Sloughed	Vacuolized
Control	97.32±1.41	1.01±0.22	0.63±0.04	1.10±0.41
75 mg/kg	96.44±1.82	1.93±0.31	0.85±0.03	1.19±0.29
375 mg/kg	93.53±0.61	2.54±1.83	2.38±1.23	1.63±0.58
750 mg/kg	74.21±0.45***	12.22±0.91*	4.29±0.41*	9.35±1.12*

Values are presented as Mean±SD for 8 mice.

* *p*<0.05,

*** *p*<0.001 compared to the Control group.

### Morphometry

The seminiferous tubules' diameters were not significantly changed in the 75 mg/kg group. The seminiferous epithelium height also did not change. In the 375mg/kg group, the seminiferous tubules' diameters and the height of the seminiferous epithelium were slightly lower than those in the control group. In the 750 mg/kg group the seminiferous tubules’ diameters(193.22±4.65; *p*=0.019) and the height of seminiferous epithelium (139.22±5.09; *p*=0.023) were significantly lower than those in the Control group (211.68±10.61; 164.31±7.11; respectively). In addition, the testis' volumes decreased in the 750 mg/kg treated mice (104.60±8.38; *p*=0.041), in comparison to the control animals (121.44±9.68). The results of the morphometric studies are shown in Table 2.

**Table 2:** Morphometric parameters in the Control and Experimental groups.

Groups	Parameter (µm)		
	STD (µm)	SEH (µm)	Testis volume (cm^3^)
Control	211.68±10.61	164.31±7.11	121.44±9.68
75 mg/kg	210.72±11.23	162.62±4.82	122.21±10.23
375 mg/kg	201.61±3.47	157.76±1.73	119.74±7.81
750 mg/kg	193.22±4.65*	139.22±5.09*	104.60±8.38*

Values are presented as Mean±SD for 8 mice.

* *p*<0.05 compared to control group. STD: Seminiferous tubule diameter, SEH: Seminiferous epithelium height.

### Spermatogenesis assessment

**Figure 2:**
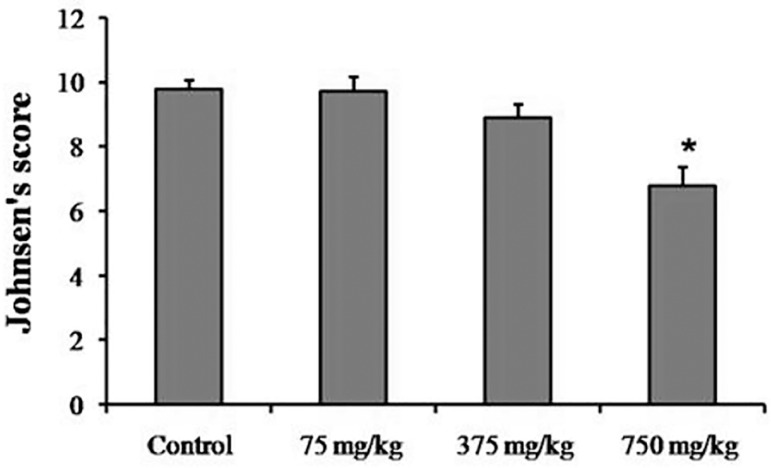
Johnsen's score in the Control and Experimental groups. Values are presented as Mean±SD for 8 mice. * *p*<0.05 compared to The Control group.

In the Control and the 75 mg/kg groups, normal spermatogenesis was observed and there was no significant difference in the mean Johnsen's scores between them. In the 375 mg/kg group, some sections contained a few tubules, in which spermatogenesis was abnormal and the mean Johnsen's score was slightly lower than those in the Control group. In the 750mg/kg group, all the sections contained a number of tubules with maturation arrest and the mean Johnsen's score was significantly lower than those in the Control group(6.87±0.65 *vs.* 9.96±0.14; *p*=0.038). The results of the mean Johnsen's score count are shown in Figure 2.

### Organ weight and testicular sperm count

As shown in Table 3, we did not find any significant change in testicle weight in the 75 and 375 mg/kg groups of treated mice. Testicle weight in 750mg/kg group was significantly decreased (0.11±0.03; *p*=0.035) in comparison to the Control group (0.14±0.02). There was no significant difference in sperm head numbers between the 75 mg/kg and the control groups. The number of sperm heads was slightly decreased in the 375mg/kg group . The sperm head count was significantly decreased in the 750 mg/kg treated animals (16.74±0.51; *p*=0.043) compared with the Control group (22.63±0.57). The results of the number of testicular sperm heads per gram of testis are depicted in Table 3.

**Table 3:** The number of testicular sperm heads per gram of testis and testicular weight in control and experimental groups.

Groups	Sperm head/testis (x10^6^)	Weight (g)
Control	22.63±0.57	0.14±0.02
75 mg/kg	22.31±0.42	0.14±0.01
375 mg/kg	20.52±0.59	0.13±0.04
750 mg/kg	16.74±0.51*	0.11±0.03*

Values are presented as Mean±SD for 8 mice.

* *p*<0.05 compared to control group.

### Testosterone Assay

**Figure 3:**
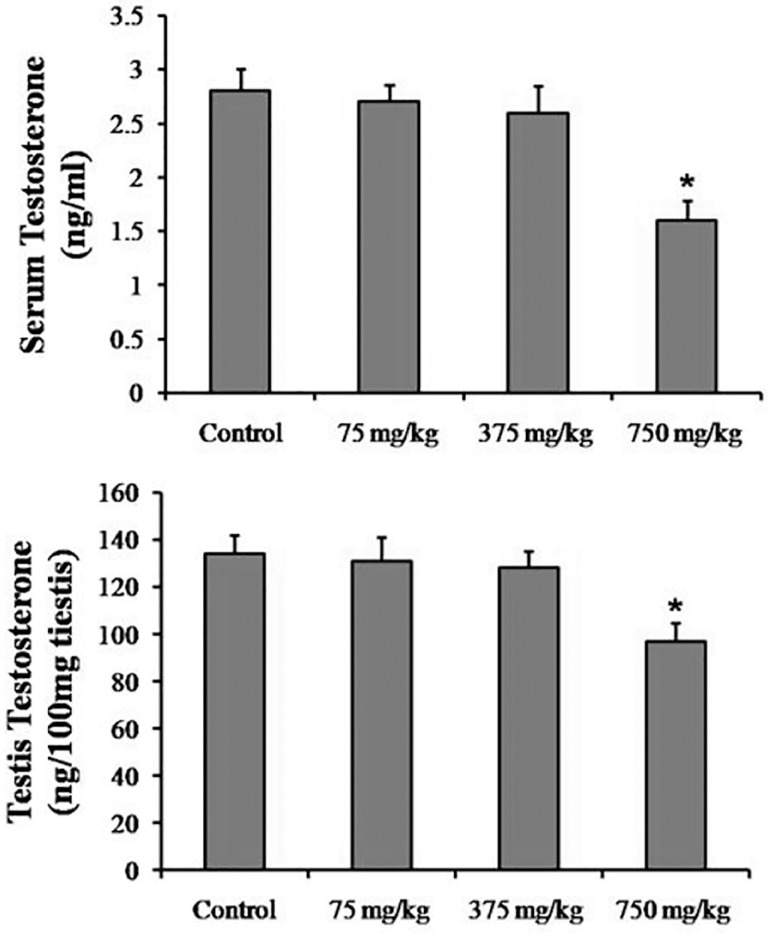
Serum and testis testosterone levels in the Control and Experimental groups. The values are presented as Mean±SD for 8 mice. *p<0.05 compared to The Control group.

As shown in Figure 3, serum and testis levels of testosterone decreased in the 750 mg/kg treated mice (1.65±0.25; *p*=0.041 and 98.83±8.67; *p*=0.034; respectively) when compared to those in the Control Group (2.81±0.32; 131.92±8.71; respectively). However, there were no significant differences in this hormone level between other groups.

## DISCUSSION

The widespread use of medicinal plants is partly due to the low toxicity attributed to these natural products (Ling *et al.,* 2008). However, medicinal plants may cause a serious toxic effect. Herbs have several chemical components that act on the whole body or some specific organ. However, some of these chemicals are mild and safe even in large doses, but some of them induce a toxic effect in large doses, or when taken continuously (Chen *et al.,* 2011). In the present study, we demonstrated that a 35- consecutive-day-treatment with 750 mg/kg extract of TM induces testicular damage in mice. Recently, another species of the same family, *Tropaeolum tuberosum*, was reported as being able to reduce testicular function in rats following treatment with extracts prepared from the roots (Cárdenas-Valencia *et al.,* 2008). In addition, it has been reported that some flavonoids, which are major components of the hydroethanolic extract of TM, are potential endocrine active compounds (Le Bail *et al.,* 1998).

With histopathology, it was possible to demonstrate changes in testis morphology, such as epithelial vacuolization, sloughing and atrophy in the mice exposed to TM. The presence of vacuoles in the Sertoli cells and the sloughing of immature germ cells from the seminiferous tubules in the 750- mg/kg group indicate that this plant might affect the Sertoli cell functions. One of the most common morphological responses of the Sertoli cell to injury is vacuolazation. In some cases, the vacuoles are large and discrete, while in others micro vacuolazation of the basal Sertoli cell cytoplasm is seen. When examined in their ultra-structure, the vacuoles most often appear to represent dilated cisternae of smooth endoplasmic reticulum, but the tortuous nature of the cytoplasmic processes of this cell often makes it difficult to determine the subcellular localization of the vacuoles (Creasy, 2001). Vacuoles have been described as an early event with many compounds, such as 2, 5-hexanedione (Chapin *et al.,* 1983), cyclohexylamine (Creasy *et al.*, 1990), 1, 3-dinitrobenzene (Blackburn *et al.,* 1988), tricresyl phosphate (Somkuti *et al.,* 1991), and phthalate esters (Creasy *et al.*, 1987).

Seminiferous tubules' diameters were significantly decreased in the 750-mg/kg-treated mice. It has been revealed that increased seminiferous tubule diameters represent fluid retention resulting from impaired emptying through the efferent ducts; whereas germ cell loss can cause decreased seminiferous tubules' diameters (Moffit *et al.,* 2007). Moreover, poor spermatogenesis in the 750 mg/kg-administrated mice was found using the Johnsen's scoring assessment. Alterations in the Johnsen's scoring relate to germ cell degeneration.

As shown in the results, the administration of 750 mg/kg of TM could significantly decrease testosterone concentrations. The suppression of testosterone was accompanied by a significant increase in histological criteria and a significant decline in testis weight, seminiferous tubule diameter and Johnsen scoring. It is known that testosterone withdrawal results in the triggering of apoptosis, as well as germ cell detachment from the seminiferous epithelium (Blanco-Rodríguez & Martínez-García, 1998). Testosterone is required to maximize the binding of round spermatids to Sertoli cells *in vitro* (Cameron & Muffly, 1991). In the testosterone- suppressed rats, elongated spermatids are absent because the round spermatids are prematurely detached from the Sertoli cells (Smith & Walker, 2014).

The toxic agents act on three main testicular target cells to disrupt the spermatogenesis included in the somatic cells, the Leydig and Sertoli cells, and the germ cells. These cell types can be selectively targeted by some specific toxicants that induce germ cell death and spermatogenic failure in animal models (Boekelheide, 2005). The main mechanism of TM effects on mouse testicular damage was not clarified in this study. The present result of immature germ cells sloughing from the seminiferous tubules indicate that this plant might affect Sertoli cell functions. An alteration in the Johnsen's scoring and morphometric data in the TM-treated mice might be associated with apoptosis induction in the testicular germ cells. In addition, the reduction in the morphometrical alterations may have been a consequence of germ cell loss. Thus, the spermatogenic defects might result, not only from a direct effect of TM on germ cell death, but also from changes to the Sertoli and Leydig cells' function.

## CONCLUSION

High doses of TM extract can change the testes' histological structure and lead to spermatogenesis failure. However, we still need future studies to clarify the mechanism of TM action on testicular tissue. Although the effects of TM extract in human reproductive activities are unknown, regarding this study, it has been suggested that infertile men, or men with reproductive disorders, must exert caution when using it during treatment.
